# ‘This restriction of expression’: migration, social catastrophes, and psychiatry in Cold War Taiwan

**DOI:** 10.1017/mdh.2025.10021

**Published:** 2025-07

**Authors:** Wen-Ji Wang, Yan-Je Yin

**Affiliations:** Institute of Science, Technology, and Society, National Yang Ming Chiao Tung University - Yangming Campus, Taipei, Taiwan

**Keywords:** Taiwan, Cold War, Cultural psychiatry, Migration, Chinese Mainlanders

## Abstract

This article explores the intersection of Cold War geopolitics, cultural psychiatry, and migration in Taiwan from the mid-1940s to the 1970s. Building on recent scholarship in cultural psychiatry and Cold War science, it examines how geopolitical tensions shaped psychiatric knowledge production in East Asia. Focusing on the psychological and social impact of the 1949 mass migration, when over a million Chinese immigrants arrived in Taiwan, alongside the clinical and academic work of Taiwanese psychiatrists, the study highlights how migration and societal upheaval became central research concerns. Operating under the authoritarian Kuomintang regime and within the constraints and opportunities of international politics, Taiwanese psychiatrists – most of whom were native-born with colonial backgrounds – drew on intellectual traditions from imperial Japan, fascist Germany, and the Cold War Western bloc. Navigating both global psychiatric discourses and local concerns, they positioned themselves as key contributors to the international development of psychiatric research. While their portrayals of Chinese character structure and family dynamics sometimes reflected essentialist views, their work also demonstrated a nuanced awareness of historical change and contemporary realities during a period of intense political repression and uncertainty. By analysing archival sources and medical texts, this article illuminates the complex interplay between geopolitics and psychiatric knowledge production in Cold War Taiwan.

On January 19, 1955, the *Keng Sheng Daily News* briefly covered the story of Man-Chiu Tien.^1^ Fleeing Communist oppression, Tien and his mother left North China for Taiwan in 1949, leaving other family members behind. Depleting all their savings after a few years, the 30-year-old, who had received higher education, managed to secure temporary employment with the state-owned Taiwan Power Company in the remote eastern part of the island. However, by the end of 1954, he had gone missing. Tien was eventually found severely mentally ill by the police. The local newspaper, stressing Tien’s hatred of the Communists and his dire economic condition, appealed to the public to assist the young man and his elderly mother with donations.^2^

After the end of the Second World War, the Chinese Nationalist government (the *Kuomintang*) took over Taiwan from Japan, which had ruled the island for half a century. However, they faced defeat in the subsequent Chinese Civil War, prompting their relocation to the island by the end of 1949. The exact number of military personnel and civilians accompanying the government to the island remains a topic of debate, with estimates ranging from one to two million.^3^ Termed as the ‘relocation to Taiwan’ or the ‘1949 Great Retreat’, this significant event had far-reaching impacts on various facets of Taiwanese life. Under the authoritarian rule of Chiang Kai-shek’s regime, compounded by ethnic tensions stemming from events like the February 28 Incident, looming attacks from the communists across the Taiwan Straits, and the immense reconstruction efforts and political transformations, the challenges of adaptation faced by the Chinese mainlanders became a central societal concern.

Over the past few decades, there has been a sustained interest in the 1949 migration. Beyond a handful of notable cultural critiques and biographical accounts, the academic realm has produced a series of works on ethnic relations and the political and social inequalities between the so-called ‘*benshengren’* (Taiwanese Han Chinese, those who migrated to the island from mainland China before the Japanese colonial period) and the ‘*waishengren’* (mainlanders, those who migrated to Taiwan from China after the Second World War).^4^ Various studies underscore the political and cultural imaginings at both individual and collective levels, alongside the evolution of ethnic consciousness.^5^ The diasporic experiences of mainlanders have also become a consistent focal point of inquiry.^6^ Among these studies, apart from a few exceptions,^7^ there exists a notable scarcity of works exploring the subject through the lens of medicine and mental health discourse. A systematic examination of Chinese incomers’ mental states and related psychiatric studies could significantly deepen our understanding of their social and psychological struggles, as well as how the emerging Taiwanese psychiatric profession interpreted these experiences within a distinct social, historical, and academic context.

The relationship between migration, disease, and health has long been a focal point of historians’ attention. The design of healthcare policies and the production of scientific knowledge by different healthcare systems raises questions about their underlying racial considerations. Additionally, the health experiences of migrants are intricately influenced by a multitude of factors and the intertwining actions of historical agents.^8^ Immigrants often find themselves facing a ‘double jeopardy’ due to migration, as they become both a minority group and afflicted by diseases.^9^ These migrants – the Irish, Chinese, and African Americans, in particular – are often viewed as the ‘other’ by the governments, healthcare systems, and broader society of their host countries.^10^ Moreover, differences among immigrants are notable, with experiences varying based on race, nationality, gender, age, and occupation.^11^ The construction of relationships between migration and mental disorders in different periods and by different types of psychiatric discourse, along with the implementation of these constructs in border controls and the allocation of government resources such as healthcare and social welfare, remains a recurring topic explored by medical historians.^12^

In recent decades, there has also been a growing interest in exploring the intersections of mental illness, psychiatry, and Cold War politics. The geopolitical tensions between the Western and Eastern Blocs and the wave of decolonisation facilitated the rapid dissemination of scientific and medical knowledge, fostering an environment where experts from former metropoles and colonies could collaborate on a relatively equal basis.^13^ During the process, Cold War politics nevertheless exerted a significant influence on the shape and substance of psychiatric knowledge.^14^ For instance, the Korean War provided a backdrop for the flourishing of military and civilian psychiatry in South Korea under American influence while also fuelling the growth of dynamic psychiatry in the United States.^15^ In both the United States and Japan, successive generations of psychological scientists pursued diverse studies on the psychological effects of nuclear warfare.^16^ Despite the pervasive Soviet influence during the communist era, Eastern European nations managed to develop psychiatric systems and methodologies imbued with national characteristics.^17^ These three examples clearly illustrate the increasing demand for a more nuanced exploration of the interplay between psychological sciences and ideology during the Cold War, foregrounding local variations and individual agency.^18^

The Cold War provides a valuable framework for the present study for several reasons. First, the United States’ foreign policy of containing communism and promoting democracy ironically led to the rise of several autocratic regimes worldwide, including the Kuomintang in Taiwan. The global Cold War tensions, combined with Chiang Kai-shek’s oppressive dictatorship, dramatically reshaped the island state, a former Japanese colony. This transformation contributed to the psychological, social, and political challenges explored in this article. Second, the considerable influence of the United States and the influx of resources from international aid agencies led to shifts in the objects of study, theoretical frameworks, and research trends across various scientific fields. For example, Taiwanese medical science was forced to grapple with sudden and substantial decolonisation in the late 1940s and early 1950s to eliminate Japanese influence and meet American standards,^19^ nevertheless creating opportunities for certain disciplines and trends to flourish. Migration and the effects of social catastrophes, for another, became important research topics at a time when social stability was a key political issue, attracting significant scholarly attention both locally and internationally. Finally, due to superpower rivalry and China’s isolation from much of the Western world until the late 1970s, Taiwan functioned, as sociologist Shao-Hsing Chen described it, as a ‘laboratory’ or ‘substitute’ for ‘the study of Chinese society and culture’.^20^ As will be discussed, Taiwanese psychiatrists actively engaged with this environment, and some emerged as experts in Chinese studies and made important contributions to the global development of cultural psychiatry. Their work on migration and culture-specific mental disorders in this sense aligns with one of the four types of Cold War social sciences outlined by Nils Gilman, which ‘arose as a result of the opportunities that the Cold War academy created’.^21^

Building on the scholarship mentioned above, the present study investigates the mental health issues relating to the mass migration in post-war Taiwan and how Taiwanese psychiatry, developing at the time, navigated the challenges posed by both this influx and Cold War politics locally and globally. Scholarly research on this topic is limited. Historian Harry Yi-Jui Wu utilises patient case notes to explore how post-war Taiwanese psychiatrists strove for professionalisation amidst Cold War tensions and social upheaval.^22^ Expanding upon this groundwork, our research aims to provide an overview of mental health issues in early post-war Taiwanese society. The mental health and social problems resulting from the mass migration were exacerbated by the authoritarian rule of the Kuomintang regime. Drawing from diverse sources, including psychiatric case files, news reports, medical literature, and autobiographical accounts, this study will analyse the ways in which psychiatric medicine, particularly from the mid-1940s to the 1970s, addressed the impact of profound societal changes, mental disorders, as well as social maladaptation among Chinese immigrants. The theoretical frameworks employed by Taiwanese psychiatrists and the research subjects they worked with were closely linked to the transnational exchange of psychiatric knowledge prior to and during this period. While actively participating in the global production of medical knowledge with a degree of academic autonomy, they had to develop strategies to grapple with sensitive issues such as ethnic tensions and turbulent social changes in a high-pressure political environment. In sum, we will argue that the geopolitical dynamics during the Cold War era not only gave rise to the mental health challenges faced by Taiwanese psychiatrists but also fostered an intellectual and social environment conducive to the development of a unique body of psychiatric knowledge.

## The psychological landscape of Chinese mainlanders

In the 1950s and 1960s, Taiwanese newspapers reported innumerable cases of suicides, mental illness, and crimes of passion, a significant portion of which involved individuals who had recently fled from China. Sun Moon Lake, a renowned tourist destination in central Taiwan, became a hotspot for suicides, drawing scores of desolate incomers.^23^ The prevalence of mental illness and disorderly vagrancy further heightened social and political instability.^24^ Additionally, crimes of passion, frequently linked to economic hardship and family discord, attracted extensive media coverage.^25^ In 1956, Dr. Hsu-chu Lee, Director of the Air Force Medical Corps, discussed the influence of Taiwan’s natural environment on mainlanders. They often experienced feelings of frustration, homesickness, excessive workloads, and susceptibility to illnesses. Additionally, abnormal mental states, including

depression and irritability, have led to a significant increase in the prevalence of mental disorders. The pervasive sense of irritability places one’s attitude toward life in a perpetual state between two extremes, swinging from left to right and vice versa. This state of flux has given rise to a multitude of incidents, ranging from suicides to various peculiar occurrences.

Lee, originally from Beijing, urged his fellow incomers to familiarise themselves with the local environment, adjust their lifestyles, and maintain their mental well-being to ‘create a new living environment and return to the mainland happily and promptly’.^26^

As pointed out by historian Yu-Chuan Wu, psychiatric disorders during the 1950s and 1960s were not merely perceived as health issues but entailed considerations of social welfare and public security. The Taiwan Provincial Government issued the ‘Taiwan Province Measures for Suppressing Deserters and Vagrants’ in 1950 to police homeless deserters, unemployed vagrants, and beggars, as well as street vendors and individuals engaging in unlawful occupations that posed threats to social order.^27^ Wu notes that managing ‘mentally ill wanderers’ became ‘one of the most important factors shaping Taiwan’s psychiatric healthcare policies during the 1950s and 1960s’.^28^ The prevalence of social turmoil, coupled with the increasing number of psychiatric patients due to the influx of incomers, prompted both central and local governments to expand, or at least plan to expand, existing social relief and psychiatric facilities, as well as establish new mental health institutions.^29^ Given the limited number of psychiatric hospitals established by central or local governments during that period and the heavy reliance on private psychiatric clinics or asylums for mental health matters, the establishment of a few facilities mainly targeting the need of Chinese incomers – the psychiatric unit at Yuli Veteran Hospital in Hualien, for instance – highlights the severity of their psychological distress.

Apart from newspaper reports and government documents, the old case notes of the Department of Neurology and Psychiatry at the National Taiwan University Hospital (hereafter as NTUH), currently stored at the Institute of Modern History, Academia Sinica, provide another invaluable glimpse into the psychological landscape of post-war Taiwan, primarily through the psychiatric lens.

A brief discussion of the historical context precedes our analysis of the case notes. During the colonial period, Taiwanese psychiatry developed primarily under Japanese physicians.^30^ Following the war, these colonial psychiatrists were repatriated, and in 1946, Tsung-Yi Lin, a 26-year-old graduate of Tokyo Imperial University, was appointed Director of the Department of Neurology and Psychiatry at the newly restructured National Taiwan University (NTU). Over the subsequent two decades, amidst shifting East Asian geopolitics and increasing Western aid, the department transitioned from Japanese and German influences to European and North American psychiatric trends. For instance, the bachelor’s dissertations completed at NTU’s Medical College in the late 1940s demonstrate a predominant focus on the hereditary and biological foundations of mental disorders.^31^ Topics of study encompassed pathobiography (e.g. Goethe, Rousseau, and Nietzsche), eugenics, and intelligence testing.^32^ While biological and neurological research continued in the 1950s and 1960s, there was a discernible shift towards incorporating social, psychological, and cultural dimensions into psychiatric inquiry.^33^ International exposure further accelerated this epistemological transformation. More than two dozen Taiwanese psychiatrists, psychologists, nurses, and social workers pursued advanced studies abroad in the 1950s and 1960s through fellowships from the World Health Organization (WHO), the US Agency for International Development, and other aid organisations. Over two-thirds trained in the US, with others studying in the UK, Canada, and Europe.^34^ Consequently, Taiwan’s early psychiatrists acquired expertise in epidemiology, cultural and dynamic psychiatry, psychotherapy, mental hygiene, and neurophysiology.^35^ Despite some psychiatrists relocating from mainland China and the military establishing its own psychiatric care system in the early 1950s,^36^ NTUH’s Department of Neurology and Psychiatry remained the most research-intensive and well-resourced institution in post-war Taiwan.

The daily practice at the department was significantly structured by the socio-political situation in post-war Taiwan. As the island’s political and social centre, Taipei attracted a substantial influx of incomers. Consequently, taking the year 1951 for instance, approximately 63% of the outpatients seeking psychiatric and neurological assistance at NTUH, at least according to the existing medical records, were from Mainland China. Many had affiliations with the military, police, and civil service.^37^ Among the myriad psychiatric and neurological cases in the same year, psychoneuroses constituted 40%, with mainlanders comprising 75% of this category. Consisting of around 16% of all cases, schizophrenia emerged as the most predominant psychosis, with the Taiwanese being more affected. Nevertheless, the paranoid subtype of schizophrenia was notably conspicuous among mainlanders.

The content of some of these medical records taken in 1951 casts light on the social, economic, and psychological suffering experienced by mainlanders in more detail, as well as how the NTUH team framed and managed their conditions. Lang, a law student at NTU, struggled with adaptation issues and was diagnosed with the anxiety state of psychoneurosis. Considering Lang’s educational background, his psychiatrist recommended that Lang write an autobiography. The college student uncovered that both his strained relationship with his father and his flawed personality were at the root of his condition. His medical record revealed that, along with medication, Lang also underwent experimental treatments such as psychoanalytic therapy.^38^ Kan, originally from a wealthy family in Shanghai, faced financial hardship upon relocating to Taiwan, leading her husband to work as a rickshaw puller. She once endured a challenging childbirth due to malnutrition. Suffering from insomnia, anxiety, and hypochondria, this mother of two was diagnosed with psychoneurosis and treated with medications.^39^ Some patients exhibited more severe conditions. While in Shanghai, a young man surnamed Teng was so troubled by nocturnal emissions and headaches that he engaged in self-harm and entertained suicidal thoughts. Recently, the 25-year-old suffered from nervousness, anxiety, and pessimism. Convinced that military trucks passing by his school were targeting him, Teng felt under constant surveillance and attempted suicide by mutilating his genitals. His diagnosis was schizophrenia, paranoid type.^40^ Kung, the 40-year-old wife of a civil servant, suffered from religious and political delusions. She believed that she was being targeted by communists who subjected her to X-ray attacks, allegedly in retaliation for her physical confrontation with Stalin. She claimed that she was later rescued by divine intervention and would receive visits from President Chiang Kai-shek. Following her immediate hospitalisation, she underwent 17 sessions of insulin shock therapy and 11 sessions of electroconvulsive therapy. Eventually, the case note indicated she was ‘moderately improved’ and discharged.^41^

A few points merit further consideration. First, as mentioned above, existing patient records show that the mass migration in late 1949 and 1950 significantly changed the ethnic composition of the patient population. The disproportionate presence of mainlanders in Taipei and its neighbouring areas created an opportunity for comparative studies among subethnic groups at NTUH. Furthermore, it very likely drew the NTUH team’s attention to migration-related issues, which will be examined in more detail in the following sections. Second, many patients exhibited symptoms before they arrived in Taiwan; some even stated that the origin of their disorders dated back a long time. For them, migration itself was a painful experience, though it exacerbated their existing social and personal suffering. On the other hand, an inclination to link current complaints with past experiences among several patients might probably result from the growing influence of a dynamic and socio-psychological style of thinking, which places particular emphasis on the critical life events that cause nervous and psychotic disorders. Third, the initial predominant usage of Japanese and German in the case notes in the mid- and late 1940s, followed by the gradual incorporation of English and Chinese, highlights the complexity of Taiwan’s medical and social history. Almost all psychiatrists who practised at the NTUH during the immediate post-war era were native Taiwanese. From affluent families with prestigious colonial educational backgrounds, they were compelled to shift their political allegiance following the island’s transfer to Chinese control, navigating profound social upheavals and linguistic shifts. These young psychiatrists had to adapt, mastering Mandarin Chinese or even the local Hokkien dialect to effectively communicate with their diverse patient population, many of whom spoke varying dialects and were from dissimilar cultural and social backgrounds. Tsung-Yi Lin and Wen-Shing Tseng aptly termed these challenges as ‘cultural shock’, reflecting the daily tribulations encountered during a transitional era.^42^

## Epidemiological studies of migration and social change

Between the late 1940s and the 1970s, the NTUH psychiatric team conducted a series of studies on mass migration and its related social changes. They can be broadly categorised into four main themes: (1) epidemiology, (2) delusions and culturally distinctive psychiatric disorders, (3) institutionalised geriatric and pauper population, and (4) suicide and other social ills. Due to word limitations, the present study will focus on the first two themes.

Between 1946 and 1948, Tsung-Yi Lin, in collaboration with Japanese instructors such as Ryosuke Kurosawa and Taiwanese public health specialist Kung-Pei Chen, spearheaded a team from the NTUH in conducting a series of epidemiological investigations on mental disorders at Baksa, Simpo, and Ampeng. Lin aimed for their findings to lay the groundwork for mental health services in Taiwan. His research methodology closely followed the approach developed by his mentor, Japanese psychiatrist Yushi Uchimura, and his team in the 1930s and 40s, which focused on racial hygiene studies conducted in Hokkaido and the Izu archipelago.^43^ Given Lin’s previous tutelage under Uchimura and subsequent responsibility for devising a mental health plan upon his return to Taiwan, this methodology choice was a logical one. In addition to census registers, the NTUH team garnered support from local authorities, police force, community leaders, medical practitioners, and schoolteachers to conduct comprehensive surveys in three townships. The total population studied during this period numbered 19,931. Published in English in 1953, these findings gained international recognition and influenced the WHO’s research in social psychiatry.^44^ The team continued to employ the same methodology to investigate the incidence of mental disorders in subsequent decades. Interestingly, Lin’s article also very briefly addressed the phenomenon of mass migration, drawing on studies by Norwegian and American scholars that examined the prevalence of mental disorders among immigrants. He attributed the rise of mental disorders in Taiwan during the early 1950s to the disruptions caused by the repatriation of Japanese nationals and the influx of Chinese immigrants.^45^

The drastic social changes and their impact on Taiwanese society became recurrent themes in the following decades. In 1961, Ming-Tso Tsuang and Hsien Rin highlighted that Taipei City’s population had grown by 500,000 after the war, more than tripling its size; a substantial proportion of these new residents were of mainland Chinese origin.

The remarkable increase in population gives rise to various societal issues related to housing, economy, education, and employment, thereby subjecting individuals to inevitable psychological stress and competition within family, school, and social life. In recent years, the Department of Neurology and Psychiatry at National Taiwan University Hospital has observed a significant manifestation of such psychological issues among patients. The number of patients’ escalation could be attributed to societal transformations.^46^

Between 1947 and 1958, Tsuang and Rin continued, outpatient attendance at NTHU saw a more than tenfold increase, primarily driven by a surge in male patients with neurotic disorders of mainland Chinese ethnicity. This rise was attributed to changes in living conditions and economic circumstances among mainlanders, particularly those in military and public service roles seeking diagnostic certificates and social insurance. Ethnicity and gender also influenced illness prevalence, with male incomers experiencing greater social and psychological stress. Mainland female patients showed a greater tendency to delusional disorders, attributed by the authors to pre-existing issues in traditional Chinese family structures and the upheaval of migration. Referencing studies on European refugees in Canada and Australia, the authors highlighted the correlation between the rise in neurotic disorders, psychophysiological reactions, and delusional disorders with the psychological impacts of shifting societal demographics.^47^

The findings from Tsuang and Rin’s 1961 article recurred in subsequent epidemiological studies in various forms and with different emphases, reflecting the impact of forced political migration, economic struggles, and ensuing maladaptation on mainlanders. Mainlanders were reported to be more prone to a variety of mental disorders, including psychophysiological reactions, neurotic disorders, and delusional reactions. Notably, middle-aged individuals, females, and those with lower socio-economic status displayed a higher prevalence of adverse psychophysiological responses to stress. Mainlander females exhibited a significant prevalence of schizophrenia, while local males faced elevated rates of depression, attributed to the psychological strain stemming from societal and economic changes. Further studies indicated that immigrants living alone, belonging to lower social classes, and residing in dormitories had the highest rates of mental disorders. Other studies suggested that many mainlanders identified strongly with Western values, such as Christianity, yet their life experiences did not align with this Westernised identity, leading to psychological maladjustment.^48^ In a 1973 comparative study on Taiwan and Japan by Hsien Rin, Carmi Schooler, and William Caudill, it was noted that, unlike Japan, where symptoms tended to be introverted, the Chinese in Taiwan exhibited distinctive symptoms like hostility, detachment from reality, anxiety, and headaches.^49^

The origin of the above-mentioned epistemological studies and how the research method was employed warrant additional examination. To begin with, as mentioned previously, the development of psychiatric medicine in Taiwan during this period was closely tied to the academic traditions of the Japanese imperial era. Uchimura’s team at Hokkaido and Tokyo Imperial Universities focused on racial hygiene and imperial medicine, conducting studies on hereditary mental illnesses and racial comparisons of mental disorders, including epidemiological research among the Ainu people in Hokkaido.^50^ Harry Wu uses the term ‘incomplete decolonisation’ to characterise this colonial legacy in post-war Taiwan.^51^ Historian Akihito Suzuki has further noted the research methods of Uchimura’s team were influenced by Swiss psychiatrist Ernst Rüdin, who was involved in planning racial policies for Nazi Germany, and his Swiss disciple Brugger. In a similar vein, Hosanna Fukuzawa not only examine the close connection between Uchimura’s work on the Ainu people and the development of eugenics and racial science in Japan and Germany but also highlights the extensive influence of the research agenda within the Japanese Empire and its impact on post-war studies.^52^ Therefore, Uchimura’s epidemiological and comparative psychiatric studies merged the attributes of ‘racial hygiene’ and ‘imperial medicine’.^53^ It is safe to say that, in terms of both research subjects and methodology, the epidemiological studies conducted by the NTUH team drew from German (including the comparativist psychiatric research with distinct colonial undertones led by Emil Kraepelin)^54^ and Japanese psychiatry. However, in the post-war international academic arena, particularly when publishing in international journals during the 1950s and 1960s, the concerns of Taiwanese psychiatrists no longer centred on racial hygiene. Even in European countries deeply influenced by German psychiatry before the war, the focus of attention had now shifted towards comparative psychiatric research focusing on cultural differences and social change.^55^ Taiwanese psychiatrists were similarly caught in this trend. This transition reflects a complex interplay between imperial Japanese, Fascist German, and Cold War West-led knowledge systems across pre- and post-Second World War eras, highlighting the complicated historical shifts in psychiatric knowledge production. It was in this academic and social milieu that the psychological impact of migration, as well as the ethnic differences in mental illness, were investigated in Taiwan.

Furthermore, the historical investigation into the link between migration and mental disorders, particularly within North American psychiatry, dates to the nineteenth century. Early studies, such as those conducted by the US Bureau of the Census, focused on immigrants’ races and their perceived impact on the native population’s mental health. Throughout the twentieth century, social scientists used statistical methods to examine how social and environmental factors affected immigrants’ susceptibility to mental disorders, with post-Second World War research focusing particularly on European refugees. Some studies indicated that immigrants had a lower proportion of mental disorders compared with the native population. Others advocated incorporating more variables (e.g. including age, gender, and education level) and delving deeper into cultural origins, migration conditions, and differences in disease types.^56^ Classic studies on foreign immigrants and migration patterns have frequently emerged in the NTUH team’s work. The team’s emphasis on the ‘distinctiveness’ or ‘breakthrough’ of its research merits further exploration. Unlike previous studies relying on hospital statistics, the NTUH team prioritised comprehensive data collection from community censuses and in-depth and sometimes intrusive interviews. The team also maintained consistency in its research methods and diagnostic systems. As they put it, this approach distinguished their research and could advance mental health efforts in developing countries.

Indeed, as Harry Wu has stated, mid-twentieth-century Taiwanese epidemiology has diverse academic origins.^57^ From the above analysis and on the foundation of studies by Hans Jakob Ritter and Volker Roelcke,^58^ Akihito Suzuki, and Hosanna Fukuzawa, it can be inferred that the early post-war Formosa Study incorporated elements from racial hygiene and imperial psychiatry from Japan and Germany, as well as the long-standing concern in North American society regarding immigrants as the ‘other’. Drastic changes in geopolitics and academic traditions prompted Taiwanese psychiatrists to make the most of an assemblage of knowledge systems they inherited and later picked up in daily practices and academic research. For instance, within the unique demographic context of Taiwan (with most residents being classified as ‘Han Chinese’ at that time), topics that might have been framed within the context of race or ethnicity in other studies were transmuted into discussions on cultural patterns and differences in stages of social change.

From another perspective, the epidemiological research conducted by the NTUH team from the mid-1940s onward addressed clinical imperatives. Tsung-Yi Lin recollected that upon returning to Taiwan after studying in Tokyo, he encountered significant challenges in communicating with patients from various ethnic groups to understand their life backgrounds, social environments, and even the normality or abnormality of their behaviours. This difficulty persisted even when interacting with local, Taiwanese-speaking farmers.^59^ On one occasion, when attempting to clarify the concept of ‘culture’ within psychiatry, Lin articulated more clearly the practical nature of cultural psychiatry research for him, stemming from the context of colonisation and the post-Second World War geopolitical landscape. He explained that such research had a tangible impact and was deeply rooted in personal life experiences.

For instance, the behavioural patterns and manifestations of mental symptoms differ among individuals who have received a Japanese education in Taiwan, those who have not received a Japanese education in Taiwan, and those who have had no connection whatsoever with Japanese education from mainland China. Thus, it can be asserted that the concept of culture or the relationship between culture and psychiatry originates entirely from the clinical context, a crucial point to underscore.^60^

The issues of cross-cultural exchange, migration, and social transformation were subjects of concern within the mainstream Western psychiatric, sociological, and anthropological circles. Naturally, they garnered the attention of Taiwan’s psychiatric elite. However, concurrently, post-war Taiwan was confronted with significant shifts in international politics, and the relevant conceptual frameworks originating from the West became essential sources of knowledge for these medical experts to understand their circumstances. Yet, delving deeper, it becomes evident that concepts such as cross-cultural experiences and migration, seemingly derived from the Western sphere, are rooted in the personal experiences of psychiatrists like Tsung-Yi Lin and Hsien Rin, who possessed colonial backgrounds and were compelled to adapt to post-colonial and Cold War political, social, and academic realities.

## Paranoia and culturally distinctive psychiatric disorders

Given their specific aims, the NTUH team’s epidemiological studies inherently offer a simplified examination of illness experiences among diverse ethnic subgroups. In comparison, their cultural and anthropological approach is evident in their more detailed analyses of the content of delusions and hallucinations and a range of culturally distinctive psychoneuroses.

The prevalence of delusional psychosis in Taiwanese society during the early post-war era captured the attention of Taiwanese psychiatrists. Simultaneously, from the 1930s to the 1960s, the enduring global interest in migration spurred the research pursuits of the NTUH team. In their collaborative article, ‘The Characteristics of Paranoid Reaction in Present Day Taiwan’, authored by Hsien Rin, Chen-Chin Hsu, and Sau-Lien Liu in 1958, they initially referenced North American studies indicating that forced immigrants often encountered mental disorders, including delusions, amidst profound cultural transitions.^61^ Despite both Taiwanese and mainlanders sharing a common overarching culture, disparities in situational backgrounds arose due to the experience of migration, leading the authors to focus on exploring the correlation between paranoid psychosis and Taiwan’s current situation. Among the four groups classified by province of origin and gender, mainland-born women exhibit the highest incidence of paranoid reaction, primarily attributed to maladjustment following migration, alongside issues like marital discord and strained relationships between mothers- and daughters-in-law. Even though mainlanders experienced long-term turbulence, their paranoid breakdowns occurred a few years after migration. The article included six case studies spanning mainland-born women, a mainland-born male, and local-born male and female patients. Employing a dynamic psychiatric approach, including psychoanalysis, the authors delved into the cases’ life trajectories and etiological mechanisms. For instance, a Fujian-born Christian woman became delusional, believing her husband was unfaithful and President Chiang was her father, anticipating a visit from a ‘prince’. Similarly, a Jiangsu-born Air Force instructor, grieving his son’s death, developed delusions, accusing his wife of sexual frigidity and suspecting colleagues of ill intentions, envisioning surveillance by secret agents. Throughout, the article highlights how migration triggered family separation, economic turmoil, and hardships, fostering grief and hostility, which in turn manifest in mental disorders such as paranoid reactions, psychoneuroses, and suicides. The authors emphasised that while interpersonal issues might aggravate these fundamental factors, Chinese culture also shapes personality structures.

In 1962, Hsien Rin, Kwang-Chung Wu, and Ching-Ling Lin conducted a study involving 220 cases, revealing that local-born patients exhibited a higher prevalence of religious, superstitious, and supernatural elements in their delusions and hallucinations. Local-born males, particularly, displayed more grandiose ideas, linked with their lower socioeconomic status. Conversely, mainlanders often expressed delusions related to political concerns, espionage, and international affairs, indicating the significant impact of migratory phenomena driven by political factors on the manifestation of delusional and hallucinatory symptoms. The findings confirmed the close connection they observed between ‘social catastrophes and the subsequent development of paranoid disorders’. ‘The type and content of delusions and hallucinations’, as the authors continued, ‘will be coloured by one’s particular experiences, personality, and cultural milieu’.^62^

As previously discussed, Western studies have shaped the exploration of migration by Taiwanese psychiatrists. Early research in the 1930s on Norwegian immigrants to the United States and African Americans migrating to New York hinted at a higher prevalence of mental disorders among migrants. However, debate persisted over whether these conditions were pre-existing or a result of migration-related challenges.^63^ By the late 1940s and early 1950s, studies on Second World War refugees relocating to Canada and Australia provided more explicit evidence of the direct link between migration and mental health. For example, Libuse Tyhurst’s study in Montreal revealed how refugees often experienced delusions of being perceived as communist spies, with anxiety manifesting through physical symptoms rather than verbal communication.^64^ Ignacy A. Listwan’s research on European immigrant patients in Australian psychiatric hospitals found that migrants, particularly young single men from Eastern Europe, were more prone to delusional symptoms, often emerging after their arrival in Australia amidst challenges such as economic hardships and loneliness. Listwan also applied Freud’s theories to elucidate migrants’ personality traits and psychological states.^65^ Moreover, migrants experienced neuroses as a form of collective anxiety arising from the psychological conflict between prejudice and assimilation. Difficulty in adaptation often led to regression and delusions, serving as coping mechanisms to exaggerate their behaviours and customs.^66^

Discussions among Taiwanese psychiatrists concerning Western research on migration from the 1930s to the 1950s reveal significant disparities in research methodologies and theoretical underpinnings. Two key characteristics emerge. First, whereas international studies primarily focused on migrants as their central analysis subjects, the NTUH team, while singling out the psychological states of Chinese incomers, directed their attention also toward major ethnic subgroups within Taiwan, conducting comparative analyses of their mental health conditions. Second, investigations into European refugees delved more deeply into migrants’ psychological states and psychodynamic mechanisms through psychoanalytic and psychodynamic lenses. In comparison, Taiwanese migration-related studies predominantly unfolded within the epidemiological framework. While these studies mirrored crucial observations by Western scholars, the depth of analysis of migrants’ psychological mechanisms in Taiwanese studies did not match that of Western ones. This disparity, however, does not stem from a lack of familiarity with psychodynamic psychiatry among Taiwanese researchers; rather, the framework of cultural psychiatry compelled figures like Hsien Rin to prioritise the interplay between traditional culture, contemporary circumstances, and individual experiences. Conversely, scholars such as Tyhurst and Listwan discussed migrants’ cultural traditions and personality traits to a limited extent, focusing primarily on migrants’ psychological responses in extreme real-world situations. Furthermore, geo-political realities or ‘the present-day situation’ loomed larger in Taiwanese psychiatric studies, so much so that the experience of migration and adaptation emerged as, at least for Taiwanese psychiatrists, the distinctive feature separating Taiwanese from mainlanders. The previously-mentioned 1958 paper on ‘The Characteristics of Paranoid Reaction in Present Day Taiwan’, for instance, underscored the contextual significance of the country’s ‘present day situation’. While emphasising the role of migration-related issues in generating paranoid symptoms among mainland patients, the authors adhered to a more traditional analytical framework when tracing the origins of conditions in Taiwanese cases to childhood family dynamics, their impact on personality development, and their longstanding supernatural beliefs. In other words, despite that the 1949 mass migration affected all ethnic groups in Taiwan, the NTUH team’s comparative psychiatric studies adopted a more sociological approach in highlighting the connection between specific forms of disease presentation, geo-political realities, and major life events among incomers, while the cases involving native Taiwanese were framed within a more cultural and, as it were, static context.

The interpretation of the entanglement between socio-political realities and disease representation took an interesting twist several decades later. Reflecting on the NTUH team’s early studies on paranoia, Hsien Rin noted in 2009 that, at least during the 1960s, compared with Taiwan, Japan exhibited a slower pace of paranoidisation among people with schizophrenia and a lower paranoid patient count. The greater prevalence of paranoia in Taiwan can be attributed to the island state’s status as a ‘modern nation formed by the coexistence of multiple ethnic groups’. At the same time, Japan has been a ‘nation with a single ethnic group and a consistent historical background’.^67^ Through this observation, Rin underscores again the role of social change, particularly modernisation (in the form of the formation of the modern state), in shaping shifts in the typology of mental illnesses. More importantly, the portrayal of ethnic diversity and turbulent historical occurrences aptly captures the erratic geopolitical, social, and psychological transformations the country has experienced.

In comparison with their earlier focus on paranoia, Taiwanese psychiatrists garnered increased international recognition through their cross-cultural examinations of culture-bound syndromes. Their investigations delved into alcoholism among aboriginal tribes, as well as phenomena such as koro, frigophobia, and neurasthenia. The latter trio, deeply rooted in Chinese cultural contexts, epitomised traditional medical and bodily conceptions.^68^ Within these discussions, issues involving migration and ethnicity surfaced subtly. The study by one of the current authors on the psychiatric discourse surrounding neurasthenia in post-war Taiwan illuminates the NTUH team’s profound engagement with the topic. This interest was intricately intertwined with several critical factors, including the surge of Chinese mainlanders into Taiwan, the burgeoning impact of traditional Chinese medicine, and the Taiwanese psychiatrists’ scholarly pursuits into Chinese cultural and character structures, coinciding with the rise of cultural psychiatry on the international stage.^69^ Taking Koro as another example, the two patients discussed in Hsien Rin’s 1963 and 1965 papers were originally from mainland China. Both were firstborn sons in conventional Chinese households, lacked maternal nurturing, and experienced a limited connection with their fathers. Economic strife and familial discord intensified anxiety and heightened sexual urges. Rin attributed their breakdowns to ‘a threat on personal and family security through employment and financial difficulties subsequent to their migration to Taiwan’.^70^ Essentially, migration exacerbated or activated existing pathological personality traits within cultural frameworks, underscoring how migration could catalyse latent pathological tendencies. Rin’s approach primarily focused on dissecting mental illness characteristics in Taiwan through cultural and comparative psychiatric lenses, thus positioning him as a cultural psychiatrist and underscoring NTUH’s stature as a prominent hub for international cultural psychiatric inquiry during that era.

The context of Hsien Rin and his colleagues’ 1975 study on frigophobia was also intertwined with the issue of migration. Among the five cases examined, four originated from various provinces of China. The authors analysed the patients’ persistent and unfounded fear of coldness through the lens of traditional medical beliefs, bodily perceptions, and Chinese familial dynamics.^71^ Although the work on frigophobia did not mention the patients’ life experiences specifically, the link between this culture-bound syndrome and significant historical events was evident to some observers. During a visit to the psychiatric wards of NTUH in the 1980s, Japanese cultural psychiatrist Koichi Ogino used his concept of ‘homelandlessness’ to understand a Chinese frigophobia patient with a history of migration.^72^

Taiwanese studies on the above-mentioned culture-bound syndromes attracted extensive international scholarly attention. Through their works, Tsung-Yi Lin, Hsien Rin, and Wen-Shing Tseng eventually became renowned researchers in cultural psychiatry. Considering that Communist China was primarily shut off from the Western world in the heat of the Cold War, as the present study has shown, Taiwanese psychiatrists capitalised on Taiwan’s status as a laboratory for Chinese studies. Their systematic inquiries into the psychological dynamics of Chinese immigrants contributed to their professional visibility at a time when global collaboration on cultural matters was actively promoted. It is also noteworthy that, during the Cold War, while many American scholars conducted fieldwork in traditional Taiwanese villages as proxies for understanding the so-called Chinese culture and history,^73^ native-born Taiwanese psychiatrists contributed to the construction of ‘Chinese Culture’ and ‘Chineseness’ through their work in comparative psychiatry, which substantially incorporated the life experiences of displaced Chinese under extraordinary historical circumstances.

## ‘ … this restriction of expression’

Five years after the lifting of Taiwan’s Martial Law in 1987, Tsung-Yi Lin commented in a speech to his fellow Taiwanese psychiatrists on the lack of comprehensive research into the psychological factors contributing to the divergence in psychiatric manifestations between Taiwanese and Mainlanders during the 1960s and 1970s. Alongside the scarcity of personnel, Lin singled out the constraints imposed by ‘a political atmosphere sensitive to enquiry into Taiwanese-Mainlander relationships’.^74^ Authoritarianism and tense ethical relationships in Cold War Taiwan were not merely the backdrop against which the NTUH team conducted their clinical work and research but constituted their everyday reality. The disappearance of Bosei Lim (or Mao-Sheng Lin) – Lin’s father and then the acting dean of the College of Humanities at NTU – during the February 28 Incident in 1947 has been considered one of the most notorious atrocities perpetrated by the Kuomintang against native Taiwanese. Eng-Kung Yeh’s father-in-law, a prominent native businessman who had been charged with seditious conspiracy, was sentenced to imprisonment for ten years. Sheng-Ji Yeh, a leftist medical student of NTU and a close friend of Hsien Rin and Eng-Kung Yeh during the immediate post-war era, noted on several occasions in his diary his disdain for mainlanders, including the authoritarian demeanour of one of his Chinese professors.^75^ Sheng-Ji Yeh ultimately faced execution due to his affiliation with an underground communist cell. A pathology professor originally from the mainland attributed the strained relationship between professors and students at NTU at the time to the undue colonial influence exerted on native students.^76^ The fact that several fellow students, professors, and even their family members perished or were significantly affected amidst the February 28 Incident and the White Terror had a substantial impact on Taiwanese psychiatrists.^77^ One of the main reasons that Tsung-Yi Lin left the country to work for the United Nations (UN) and later in Canada was the death of his father and the oppressive political environment. Eng-Kung Yeh, Tsu-Pei Hung, and Chu-Chang Chen mentioned their experiences of the White Terror in their biographies, and the former championed native consciousness vocally later in his life.^78^ American psychiatrist and anthropologist Arthur Kleinman gives a vivid description of the ‘political tensions and historical resentments’ he and his wife witnessed when they visited Taiwan in the 1970s. Due to the dominance of the ‘authoritarian mainland governing elite’, the young native psychiatrists and nurses with whom Kleinman collaborated at NTUH harboured clandestine support for political independence.^79^ This sentiment was also observed by several American scholars who conducted research in Taiwan during the Cold War era.^80^

Despite the NTUH team’s exploration of narcoanalysis in forensic investigations and the involvement of certain members in overseeing mentally ill political prisoners under intelligence agencies,^81^ their academic trajectories suggest a degree of autonomy or agency. In the immediate postwar period, when the Kuomintang regime began implementing a series of decolonisation policies, the Japanese influence in psychiatry remained significant. Furthermore, from the early 1950s onward, their research topics displayed both diversity and continuity, with a number of studies addressing politically sensitive issues such as ethnic relations and social maladaptation. In this sense, the present study corroborates the findings of Harry Yi-Jui Wu on the professional agency of Taiwanese psychiatrists during the Cold War period.^82^ This partial ‘autonomy’, at least from the authoritarian Kuomintang regime, was nevertheless also a product of Cold War geopolitics. A substantial part of the funding for training psychiatric professionals and establishing mental health services in early post-war Taiwan was provided by international aid agencies such as the WHO, the American Bureau of Medical Aid to China, and the United States Agency for International Development.^83^ Nevertheless, exploring the psychological landscape of the Cold War-era Taiwan and the impacts of social catastrophes still posed a significant challenge for Taiwanese psychiatrists. Some of the NTUH team members managed to navigate the limitations imposed on them. Sharing the result of their cultural and psychological investigations, sometimes coupled with social critique, through international journals was one of the options, and the following two examples illustrate the point.

One of the major studies conducted by the NTUH team in the 1940s and 1950s was the socio-psychomatic study of hypertension.^84^ As Tsung-Yi Lin explained, the project was closely linked with the experience of mass migration: ‘The rapid social change after the influx of mainland Chinese since 1948 led to the postulation of a possible association between the apparent increase of hypertensives and socio-environmental factors’.^85^ Data from around 10,000 hypertensives in Taipei, comprising both Taiwanese and Mainlanders, were gathered to determine the frequency and distribution of hypertension among ethnic Chinese and the relationships between biological and socio-psychological factors and hypertension. Published in Chinese in the *Journal of the Formosan Medical Association* in 1956, the first report of the study only provided preliminary statistics.^86^ No following report can be found in the Taiwanese journal, and another report published in *Clinical Science* several years later reiterated the initial study’s statistical findings.^87^ Tsu-Pei Hung, one of the co-authors of these two reports, published a follow-up study in Japanese in *the Japanese Circulation Journal* in 1961.^88^ Contrary to the widespread belief that mainlanders, due to migration and adaptation pressure, might experience higher blood pressure, the findings of Hung and the NTUH team revealed otherwise. Across gender and social class, hypertension incidence was predominantly higher among the local population. Drawing upon psychodynamic and psychosomatic studies by Franz Alexander, Leon J. Saul, and Stewart Wolf, he concluded that mainlanders often channelled their hostility and aggression through projection. This discovery aligned with the NTUH team’s earlier findings, indicating that mainlanders, compared with Taiwanese, were more prone to paranoid psychosis. Moreover, Hung continued, the escalating instances of suicides, forced suicides, and antisocial crimes among mainlanders were also linked to this outward expression of hostility. While highlighting the aggression of the mainlanders, the Taiwanese neurologist intimated a more subdued way by which the Taiwanese faced social changes and political oppression. Interestingly, Saul’s study was grounded in seven psychoanalytic cases, placing primary emphasis on the dynamics between patients and their parents alongside personality development traits.^89^ Conversely, Hung, resonating with Alexander’s work on the psychological distress of African Americans, redirected focus from individual analysis to exploring collective psychology within subcultures or ethnic subgroups.

Another instance of Taiwanese psychiatrists utilising international platforms to voice their discontent is related to their research on child mental health. Eng-Kung Yeh characterised the post-war political regime transition as ‘a state of social confusion’, highlighting that the significant social change since 1948 had led to ‘social tension and conflicts in the traditional ways of life and security system’. In addressing the problem of so-called ‘low intelligence’ and ‘poor school records’ among some schoolchildren, he reframed the issue. He attributed its origin to overpopulation, the implementation of compulsory education, and the lack of sufficient schools.^90^ Referring to a similar issue, Tsung-Yi Lin recounted in 1961 the experience of one of his colleagues who endeavoured to develop a standardised intelligence test in the late 1940s. With the implementation of government policy on the limited use of the Japanese language, the psychologist discovered that schoolchildren participating in the experiment faced difficulties in articulation, describing concrete objects, and engaging in abstract thinking.

It was thought at first that this might have been due to sampling bias … , but it became clear, by retesting some of the earlier subjects, that the enforced use of a new language, Mandarin Chinese, in the school was responsible for *this restriction of expression* [emphasis added].

The research project was then temporarily suspended ‘until new patterns of communication and culture in general had established themselves in the community’.^91^ As evidenced by the present study, for these Taiwanese elites, this ‘restriction of experience’ was a ‘cultural’ phenomenon affecting a considerable portion of the population, including themselves, and was not confined to the native schoolchildren who were compelled to undergo speedy decolonisation.

## Conclusion

In chronicling the development of cultural psychiatry in the second half of the twentieth century, recent scholarship has focused on the field’s aspirational pursuit of ‘common humanity’ amidst the backdrop of decolonisation. This was notably evident in the concerted efforts of cultural psychiatry within Western Europe and North America to disentangle itself from colonial legacies. Examining the psychological landscape of Chinese mainlanders and their portrayal by Taiwanese psychiatrists, the present study underscores the importance of Cold War geopolitics on the production of psychiatric knowledge in East Asia. The rivalry between the Western and the Communist blocks and the relocation of the Kuomintang regime to the island not only exacerbated the existing social and psychological distress among Taiwan’s different ethnic groups but also fostered a unique environment conducive to a nascent body of psychiatric knowledge. Migration and societal upheavals, prevalent mental health concerns in the West, emerged as enduring and, in some cases, primary focal points of research for the NTHU team over three decades. The growing international interest in social understanding of personality formation and mental disorders drew Taiwanese psychiatrists’ attention to the dire social and political conditions, the consequences of which they were compelled to address. Leveraging available epistemological resources from imperial Japan, fascist Germany, and the Cold War Western bloc, they researched issues that were simultaneously global and local concerns, navigating sensitive terrain with a nuanced approach. Through investigations into epistemology, ethnic variations in manifestations of paranoia, culture-bound syndromes, and related matters, the team positioned itself as a pivotal hub for international collaboration when China remained largely inaccessible to much of the world. Rather than being conduits for prevailing social and racial biases, these psychiatric professionals, primarily originating from elite local Taiwanese families with colonial backgrounds, were active but discreet agents in knowledge production amidst socio-political constraints and opportunities. In this sense, the present study resonates with recent scholarship on Cold War social sciences, which highlights local contexts and individual agency in knowledge production during a period of intense geopolitical and ideological conflicts.^92^

As the present study has shown, Taiwanese study of and reference to the 1949 migration was primarily considered within the framework of comparative psychiatry or psychiatric epidemiology, rather than directly addressing the issue from the perspectives of personal traumatic experience, social and cultural assimilation, or ethnic tensions as was more common in Western academic circles at the time. The impact of the influx of millions of Chinese incomers also appeared in various forms in other fields of their study, including migration itself, culture-bound syndromes, hypertension, and other topics not covered in the article. We argue that this seemingly circuitous or tactful probing of the matter, with a particular emphasis on the cultural and sociological exploration of the psychological landscape of mainlanders, does not diminish the importance of migration for the Taiwanese psychiatrists; rather, given that the 1949 migration reshaped nearly every aspect of Taiwanese society, the extensiveness of its impact was such that the production of psychiatric knowledge during the period took on a distinctive feature. The characteristic perspective of Taiwanese psychiatrists on the connection between migration and mental disorders was shaped primarily by their clinical interest, practical concerns, and academic lineage, but, as Tsung-Yi Lin suggested, it may have also been influenced by the ethnic tension, social disruption, and political suppression that were part of their daily reality.

The social and political realities significantly influenced both the work of Taiwanese psychiatrists and their personal lives. Some of their portrayals of Chinese character development often leaned towards essentialism and schematic representations, emphasising the influence of traditional folk concepts and oedipal relationships prevalent within Chinese families. Nevertheless, the recurrent references to ‘present day situation’ or ‘change of circumstances’^93^ in their work indicate a keen awareness of the impact of major historical events on shaping ethnic differences in illness presentation and personality structure. This awareness later translated into actions, which shaped their subsequent careers. Some of them, having already undergone significant identity shifts, ultimately saw migration as a viable option. Indeed, several prominent members of the NTHU team eventually relocated to North America, mirroring the broader migration patterns of their compatriots during an era of brain drain. In an era of political repression and uncertainty, migration was not merely an academic topic examined through the suffering of others but a personal reality that they were compelled to confront and navigate in their own lives. The title of Wen-Shing Tseng’s intellectual autobiography, ‘*One Life, Three Cultures: The Self-Analysis of the Impact of Chinese, Japanese, and American Cultures on My Personality Formation*’, aptly captures the interconnections between migratory experience, identity politics, socio-political realities, and knowledge production inherent in Cold War Taiwan.^94^

## Footnotes

1 An earlier version of this article was presented at the 43^rd^ International Conference on Chinese Studies, Chung-Ang University, Seoul, South Korea on August 18^th^, 2023. We would like to thank Kyu-Hwan Sihn and other participants in the panel for their comments. Special thanks also go to the reviewers for their insightful suggestions. This research received funding from the National Science and Technology Council, Taiwan (NSTC 113-2410-H-A49-048-MY2).

2 ‘Tonghen feibang baoxing Luji qingnian shenjingshichang rexinren she kai shen yuanshou [Detesting Communist Violence, Mentally Unstable Youth from Shandong Receives Support from the Kind-hearted]’, *Gengsheng Bao*, 19 January 1955.

3 Dominic Meng-Hsuan Yang, *The Great Exodus from China: Trauma, Memory, and Identity in Modern China* (Cambridge: Cambridge University Press, 2020): 76–80; Ko-hua Yap, ‘Cong jiemi dangan zhonggu erzhan hou yiru Taiwan de waishengji renshu [Reassessing Number of Mainland Chinese Immigrants with Declassficaited Archival Data]’, *Taiwan Historical Research*, 28 (3) (2021), 211–29.

4

Dominic Meng-Hsuan Yang and Mau-Kuei Chang , ‘Understanding the Nuances of *Waishengren*: History and Agency’, China Perspectives, 3 (2010): 108–22.

5

Mau-Kuei Chang (ed.): Guojia yu Rentong Yixie Waishengren de Guandian [*Nation and Identity: Perspectives of some ‘Waishengren’*] (Taipei: Socio Publishing, 2010).

6

Tai-Li Hu , ‘Yuzi yu fanshu: Taiwan rongmin de zuqun guanxi yu rentong [Taros and Sweet Potatoes: Ethnic Relations and Identities of “Glorious Citizens” (Veteran mainlanders) in Taiwan]’, Bulletin of the Institute of Ethnology Academia Sinica, 69 (1990): 107–32; Antonia Chao , ‘Daizhu Caomao Daochu Lüxing Shilun Zhongguo Liuwang Nüxing Zhuti yu Jiyi jian de Jiangou Guanxi [A Forever Banished Uterus: Chinese Diaspora, Fertility, and the Politics of Suffering]’, Taiwan: A Radical Quarterly in Social Studies, 41 (2001): 53–97 ; Kuang-Chun Li (ed.): Ri yu Ku: Zhanzheng de Yanxu [*Sufferings of Waishengren under the War Regime*] (Taipei: Socio Publishing, 2010).

7

Kuan-Hsing Chen , ‘Chen Yingzhen de disan shjjie kuangren/fengzi/jingshenbing pian [Chen Yingzhen’s Third World on Lunatics, Madman, Mental Patient]’, Taiwan: A Radical Quarterly in Social Studies, 78 (2010): 215–68; Shu-Chun Li , ‘Yanshuo zhi kunjing yu jia/guo “rong yu zhe”:Lun Hu Shu-wen de baise kongbu shuxie yu zhengzhi pipan [The Dilemma of Speaking and the Redundant of Home/State: Hu Shu-wen’s White-Terror Writing and Political Criticism]’, Bulletin of Taiwanese Literature, 36 (2020): 53–92 ; Hung-Bin Hsu , ‘Redai huanjing, yiju zhe yu mingan shenti: 1980 nian zhiqian Taiwan guomin yanjiu yu zhenliao [Tropical Environment, Immigrant Population, and Sensitive Bodies: Research on Allergy and Treatment in Taiwan before 1980]’, Taiwanese Historical Research, 23, 2 (2016): 125–74.

8

Lara Marks and Michael Worboys (eds.): Migrants, Minorities and Health: Historical and Contemporary Studies (London: Routledge, 1997); Alison Bashford (ed.): Medicine at the Border: Disease, Globalization and Security (Houndmills: Palgrave and Macmillan, 2006); Catherine Cox and Hilary Marland (eds.): Migration, Health and Ethnicity in the Modern World (Basingstoke: Palgrave Macmillan, 2013).

9 Lara Marks and Michael Worboys, ‘Introduction’, in Marks and Worboys, *op. cit.* (note 8): 6.

10 Desmond Manderson, ‘”Disease, Defilement, Depravity”: Towards an Aesthetic Analysis of Health. The Case of the Chinese in nineteenth-century Australia’, in Marks and Worboys, *op. cit.* (note 8): 22–48; Liam Greenslade, Moss Madden and Maggie Pearson, ‘From Visible to Invisible: The “Problem” of the Health of Irish People in Britain,’ in Marks and Worboys, *op. cit.* (note 8): 147–78.

11 Catherine Cox and Hilary Marland, ‘Introduction,’ in Cox and Marland, *op. cit.* (note 8): 5.

12

Ian Robert Dowbiggin , Keeping America Sane (Ithaca: Cornell University Press, 1997); Alison Bashford, ‘Insanity and Immigration Restriction’, in Cox and Marland, *op. cit.* (note 8): 14–35.

13

Matthew Heaton , Black Skin, White Coats: Nigerian Psychiatrists, Decolonization, and the Globalization of Psychiatry (Athens: Ohio University Press, 2013)10.1080/13648470.2015.109660427454564; Harry Yi-Jui Wu , Mad by the Millions: Mental Disorders and the Early Years of the World Health Organization (Cambridge, Massachusetts: The MIT Press, 2021).

14

Ana Antić , ‘Transcultural Psychiatry: Culture Difference, Universalism and Social Psychiatry in the Age of Decolonisation’, Culture, Medicine and Psychiatry, 45 (2021): 359–8433905076 10.1007/s11013-021-09719-4PMC8437902; Ana Antić , ‘Decolonizing Madness? Transcultural Psychiatry, International Order and Birth of a “Global Psyche” in the Aftermath of the Second World War’, Journal of Global History, 17, 1 (2022): 20–41 ; Wu, *op. cit.* (note 13).

15

Jennifer Yum-Park , ‘“Yankee-Style Trauma”: The Korean War and the Americanization of Psychiatry in the Republic of Korea’, in Mark S. Micale and Hans Pols (eds.) Traumatic Past in Asia: History, Psychiatry, and Trauma from the 1930s to the Present (New York: Berghahan Books, 2021): 96–127 ; Ji-Hye Shin and Sung-Vin Yim , ‘A Foundation for a “Cheerful Society”: The Korean War and the Rise of Psychiatry’, Korean Journal of Medical History, 32 (2023): 553–9137718562 10.13081/kjmh.2023.32.553PMC10556413; In-sok Yeo , ‘Psychiatric Casualties during the Korean War: Focusing on American and Common Wealth Soldiers’, Korean Journal of Medical History, 32 (2023): 865–8938273723 10.13081/kjmh.2023.32.865PMC10822695.

16

Ran Zwigenberg , ‘Healing a Sick World: Psychiatric Medicine and the Atomic Ages,’ Medical History, 62, 1 (2018): 27–49 29199929 10.1017/mdh.2017.75PMC5729876.

17

Mat Savelli and Sarah Marks (eds.): Psychiatry in Communist Europe (Basingstoke: Palgrave Macmillan, 2015).

18

Mat Savelli , ‘Beyond Ideological Platitudes: Socialism and Psychiatry in Eastern Europe’, Palgrave Communications, 4, 45 (2018): 1–8 ; Ana Antić , Non-Aligned Psychiatry in the Cold War: Revolution, Emancipation and Re-Imaging the Human Psyche (Basingstoke: Palgrave Macmillan, 2021).

19

Michael Shiyung Liu , ‘Transforming Medical Paradigms in 1950s Taiwan’, East Asian Science, Technology and Society: An International Journal, 11, 4 (2017): 477–97.

20

Shao-Hsing Chen , ‘Taiwan as a Laboratory for the Study of Chinese Society and Culture’, Bulletin of the Institute of Ethnology, 22 (1966): 1–14 .

21

Nils Gilman , ‘The Cold War as Intellectual Force Field’, Modern Intellectual History, 13, 2 (2016): 507–23; Mark Solovey and Christian Dayé , ‘Introduction: Cold War Social Science, Transnational Entanglements’, in Mark Solovey and Christian Dayé (eds.) Cold War Social Science: Transnational Entanglements (Cham: Palgrave Macmillan, 2021): 1–41 .

22

Harry Yi-Jui Wu , ‘A Charted Epidemic of Trauma: Case Notes at the Psychiatric Department of National Taiwan University Hospital between 1946 and 1953’, in Howard Chiang (ed.). Psychiatry and Chinese History (London: Pickering & Chatto, 2016): 160–82.

23 ‘Liu shu cheng zisha quhou yaowuzong [Left Suicide Note, Youth Disappeared without a Trace]’, *United Daily*, 18 April 1953.

24 ‘Aiguo de jingshenbing huanzhe [Patriotic Mental Patient]’, *China Times*, 17 April 1956.

25 ‘Yi qi buzhen dangjie xingxiong hongzhuan zading zhongshang zhisi [Suspected Infidelity Leads to Fatal Street Attack: Victim Fatally Injured by Brick Assault]’, *United Daily*, 5 November 1961; ‘Hong zhuanshaqiming anbei gaohuomiansi zuiliuxingbaogai panwuqixing [Brick Murder Case: Defendant Acquitted of Death Penalty, Liu Xingbao Sentenced to Life Imprisonment]’, *United Daily*, 26 May 1963.

26 Zong-zhou Liu, ‘Dalu laitai renshi de ziran diren: Duofa jibing de jiantao [The Natural Enemies of Mainlanders in Taiwan: On Multiple Diseases]’, *United Daily*, 5 July 1956.

27 Shu-Yun Liu, ‘Cong jingzheng dao shezheng: Taibeishi youmin shourong zhengce zhi bianqian [From Police Affairs to Social Work: Transformation of Taipei City’s Policy on Homeless Sheltering]’ (unpublished MA thesis: Shih Hsin University, 2010); Fang-Hsun Chan, ‘Anzhi haishi Xianzhi? Taibeishi nanxing youmin shourong znzhi jingyan zhi tantao [Settlement or Restriction ? The Shelter and Assistance Service Experiences of Homeless Men in Taipei City]’ (unpublished MA dissertation: National Chengchi University, 2017).

28

Yu-Chuan Wu ‘Zhuanye guojia meiyuan yu minjian shehui: 1945-1970 nian Taiwan de jingshen yiliao [The Profession of Psychiatry, the State, U.S. Aid, and Civil Society: Mental Health Care in Taiwan, 1945-1970]’, New History, 33, 3 (2022): 87–150 .

29 ‘Taizhong jingshen bingyuan kuojian bingfang jungong [Expansion of Wards at Taichung Mental Hospital Completed]’, *Central Daily News*, 30 August 1953; ‘Yiliao mafengbing ji jingshenbing: Tuichuyi guanbing fudaohui niding jihua jijiang shishi [Veterans Affairs Council to Implement Medical Plans for Leprosy and Mental Illness]’, *Central Daily News*, 15 March 1956; ‘Mei dui wo rongmin yiyao yuanzhu xia Rongmin zongyiyuan zuo dianji [With U.S. Aid, Groundbreaking Ceremony Held Yesterday for Veterans General Hospital]’, *United Daily*, 7 June 1956; ‘Jingshen binghuan zhe fuyin: Kaohsiung jingshenbingyuan yuenei zhaobiao xingjian [Good News for Mental Patients: Kaohsiung Mental Hospital to Commence Construction with Bidding within a Month]’, *Central Daily News*, 22 July 1959; ‘Beishifu ze zhi Wufenpu choujian shili jingshen bingyuan [Taipei City Government Chooses Wufenpu Site: Planning to Construct Municipal Psychiatric Hospital]’, *United Daily*, 6 April 1962.

30 Yu-Chuan Wu, ‘Bingtai de minzu: Rizhi wanqi Taiwan de minzuxing jingshenbing [“Pathological” Races: A History of Ethnical Mental Illnesses in Late Colonial Taiwan]’ (unpublished MA dissertation: National Tsing Hua University, 2005).

31

Hsien Rin (ed.) Guoli Taiwan Daxue Yixueyuan Fushe Yiyuan Shenjingjingshenke Nianwu Zhounian Jiniankan [*25th Anniversary Commemorative Publication of the Neuro-Psychiatry Department at the National Taiwan University Hospital*] (Taipei: National Taiwan University Hospital, 1972): 150; Chu-Chang Chen , Yongheng Zhi Chun [*Forever Spring: Chu-Chang Chen’s Memories from the Medical Field*] (Taipei: Chu-Chang Chen, 2014): 40 .

32

Bernhard Leitner , ‘For Body, Mind and the Nation: An Archaeology of Modern Japanese Psychiatry’, in Rudiger Frank , Ina Hein , Lukas Pokorny and Agnes Schick-Chen (eds.) Vienna Journal of East Asian Studies, 5 (Vienna: Praesens Verlag, 2014): 111–38.

33 As Hsien Rin later recalled, residents at NTUH in 1950 engaged with the works of Karl Menninger, Karen Horney, and Lawrence Kubie in Japanese translation, alongside standard psychiatric texts by David Henderson and Lawrence Kolb. Hsien Rin, interviewed by Wen-Ji Wang, October 2004.

34 Rin, *op. cit.* (note 31): 97.

35

Tsung-Yi Lin , Jingshen Yixue zhi Lu: Hengkua Dongxi Wenhua [*The Path of Psychiatry: Bridging Eastern and Western Cultures*] (Taipei: Daw Shiang Publishing, 1990): 65–75 .

36

Ting-She Chang , Shih-Mao Lo , and Dong-Sheng Tzeng , ‘Sanjun zongyiyuan Beitou fenyuan zhi jinxi yu Taiwan jingshenyixue fazhan shi [The Past and Present of Tri-Service General Hospital Beitou Branch: A History of Psychiatry Development in Taiwan]’, Taiwan Medical Journal, 60, 5 (2017): 278–81.

37

Wen-Ji Wang , ‘Managing Chineseness: Neurasthenia and Psychiatry in Taiwan in the Second Half of the 20^th^ Century’, History of Psychiatry, 33, 3 (2022): 263–7835466754 10.1177/0957154X221087410.

38 Case number 1951/190, Department of Neurology and Psychiatry, NTUH. The names of the patients mentioned in this article have been changed to maintain confidentiality.

39 Case number 1951/873, Department of Neurology and Psychiatry, NTUH.

40 Case number 1951/655, Department of Neurology and Psychiatry, NTUH.

41 Case number 1951/595, Department of Neurology and Psychiatry, NTUH.

42 Wang, *op. cit.* (note 37).

43 Lin, *op. cit.* (note 35): 21; Hsien Rin, interviewed by Wen-Ji Wang, October 2004. For Tsung-Yi Lin’s prewar academic training and its impact on the development of postwar Taiwanese psychiatry, see also Alex Hsu-Chun Liu, ‘Translating Postcolonial Mental Landscapes: Hsien Rin in the Making of Transcultural Psychiatry and Psychological Anthropology of Taiwan and the World, 1950-1980’ (unpublished MA dissertation: National Yang Ming Chiao Tung University, Taiwan, 2025).

44

Harry Yi-Jui Wu , ‘From Racialization to World Citizenship: The Transnationality of Taiwan and the Early Psychiatric Epidemiological Studies of the World Health Organization’, East Asian Science, Technology, and Society: An International Journal, 10, 2 (2016): 183–205 .

45

Tsung-Yi Lin , ‘A Study of Incidence of Mental Disorder in Chinese and Other Cultures’, Psychiatry, 16, 4 (1953): 313–3613134403 10.1080/00332747.1953.11022936.

46

Ming-Tso Tsuang and Hsien Rin , ‘Daxue yiyuan jingshenke menzhen bingren zhi shehui ji renkou bianqian yinsu zhi tantao [An Evaluation of Social and Migration Factors among Psychiatric Outpatients in the University Hospital]’, Journal of Formosan Medical Association, 60, 1 (1961): 30–5, 30 13778414.

47 Tsuang and Rin, *op. cit.* (note 46): 34.

48

Hsien Rin , Hung-Ming Chu and Tsung-Yi Lin , ‘Psychophysiological Reactions of a Rural and Suburban Population in Taiwan’, Acta Psychiatrica Scandinavica, 42, 4 (1966): 410–735964946 10.1111/j.1600-0447.1966.tb06178.x; Tsung-Yi Lin , Hsien Rin , Eng-Kung Yeh , Chen-chin Hsu , and Hung-Ming Chu , ‘Mental Disorders in Taiwan, Fifteen Years Later: A Preliminary Report,’ in William Caudill and Tsung-Yi Lin (eds.): Mental Health Research in Asia and the Pacific (Honolulu: East-West Center Press, 1969): 66–91 ; Norman A. Chance , Hsien Rin , and Hung-Ming Chu , ‘Modernization, Value Identification, and Mental Health: A Cross-Cultural Study’, Anthropologica, 8, 2 (1966): 197–216 ; Hung-Ming Chu , ‘Migration and Mental Disorders in Taiwan’, in William P. Lebra (ed.): Transcultural Research in Mental Health (Honolulu: The University Press of Hawaii, 1972): 275–325 ; Hsien Ri , ‘Jingshenyixue yu Shehui (VI): Qianxi yu Jingshen jihuan [Psychiatry and Society (VI): Migration and Mental Illness]’, Medicine Today, 3, 6 (1976): 538–42.

49

Hsien Rin , Carmi Schooler , and William A. Caudill , ‘Culture, Social Structure and Psychopathology in Taiwan and Japan’, Journal of Nervous and Mental Disease, 157, 4 (1973): 296–312 4729065 10.1097/00005053-197310000-00009.

50 Lin, *op. cit.* (note 35): 21; Wen-Shing Tseng , Handbook of Cultural Psychiatry (San Diego: Academic Press, 2001): 199–200 .

51

Harry Yi-Jui Wu , ‘Taming the Tropics with Numbers: The Origins of Psychiatric Epidemiology in Colonial Taiwan’, in Anne M. Lovell and Gerald M. Oppenheimer (eds.): Reimaging Psychiatric Epidemiology in a Global Frame: Toward a Social and Conceptual History (Woodbridge: Boydell & Brewer, 2022): 283–306 .

52

Hosanna Fukuzawa , ‘Creating Racial Subjects: Eugenics, Psychiatry, and the Ainu’, Critical Asian Studies, 56, 2 (2024): 277–96.

53

Yushi Uchimura et al., ‘Tōkyō fuka Hachijōjima jūmin no hikaku seishin igaku-teki narabini iden byōrigaku-teki kenkyū [A Comparative Psychiatric and Genetic Pathological Study on Residents of Hachijojima Island, Tokyo Prefecture]’, Psychiatria et Neurologia Japonica 44 (1940): 745–82; Yushi Uchimura et al., ‘Tōkyō fuka Miyakejima jūmin no hikaku seishin igaku-teki narabini iden byōrigaku-teki kenkyū [A Comparative Psychiatric and Genetic Pathological Study on Residents of Miyake Island, Tokyo Prefecture]’, Minzoku Eisei, 10, 1–2 (1942): 1–9; Akihito Suzuki , ‘Psychiatric Surveys and Eugenics in the Family and Community in Japan 1935-1945’, in Thomas Müller (ed.): Zentrum und Peripherie in der Geschichte der Psychiatrie: Regionale, Nationale und Internationale Perspektiven (Stuggart: Franz Steiner Verlag, 2017): 189–201 .

54

Eric J. Engstrom and Ivan Crozier , ‘Race, Alcohol and General Paralysis: Emil Kraepelin’s Comparative Psychiatry and His Trips to Java (1904) and North America (1925)’, History of Psychiatry, 29, 3 (2018): 263–8129860873 10.1177/0957154X18770601.

55

Yoshibumi, Nakane , Yasuyuki Ohta , and Mark H. B. Radford , ‘Epidemiological Studies of Schizophrenia in Japan’, Schizophrenia Bulletin, 18, 1 (1992): 75–84 1553505 10.1093/schbul/18.1.75; Erik Strömgren , ‘Recent History of European Psychiatry—Ideas, Developments, and Personalities’, American Journal of Medical Genetics, 54 (1994): 405–107726216 10.1002/ajmg.1320540422.

56

H. B. M. Murphy , ‘The Low Rate of Mental Hospitalization Shown by Immigrants to Canada,’ in Charles Zwingmann and Maria Pfister-Ammende (eds.), Uprooting and After… (New York: Springer Verlag, 1973): 221–31; H. B. M. Murphy , ‘Migration, Culture and Mental Health’, Psychological Medicine, 7 (1977): 677–84594247 10.1017/s0033291700006334; Allan V. Horwitz and Gerald N. Grob , ‘The Checkered History of American Psychiatric Epidemiology’, The Milbank Quarterly, 89, 4 (2011): 628–5722188350 10.1111/j.1468-0009.2011.00645.xPMC3250636; Eureka Henrich , ‘Constructing the “New Australian Patient”: Assimilation as Preventative Medicine in Postwar Australia’, Histoire sociale/Social History, 52, 105 (2019): 109–3531231139 10.1353/his.2019.0005PMC6588523.

57 Wu, *op. cit.* (note 51): 302–5.

58

Hans Jakob Ritter and Volker Roelcke , ‘Psychiatric Genetics in Munch and Basel between 1925 and 1945: Programs-Practices-Cooperative Arrangements’, Osiris, 20, (2005): 263–8820503766 10.1086/649421.

59 Lin, *op. cit.* (note 35): 7.

60 Lin, *op. cit.* (note 35): 214.

61

Hsien Rin , Chen-Chin Hsu and Sau-Lien Liu , ‘The Characteristics of Paranoid Reaction in Present Day Taiwan’, Memoirs of the College of Medicine of the National Taiwan University, 5, 1–2 (1958): 1–15 .

62

Hsien Rin , Kwang-Chung Wu and Ching-Ling Lin , ‘A Study of the Content of Delusions and Hallucinations manifested by the Chinese Paranoid Psychotics’, Journal of Formosan Medical Association, 61, 1 (1962): 46–57 14492322.

63

Oernulv Oedegaard , ‘Emigration and Insanity’, Acta Psychiatrica et Neurologica Scandinavica. Supplementum, 4 (1932): 1–206 .

64

Libuse Tyhurst , ‘Displacement and Migration: A Study in Social Psychiatry’, American Journal of Psychiatry, 107 (1951): 561–814819341 10.1176/ajp.107.8.561.

65

Ignacy A. Listwan , ‘Paranoid States: Social and Cultural Aspects’, Medical Journal of Australia, 1, 19 (1956): 776–813333426 .

66

Ignacy A. Listwan , ‘Mental Disorders in Migrants: Further Study’, Medical Journal of Australia, 46, 17 (1959): 566–813656032 10.5694/j.1326-5377.1959.tb59254.x.

67

Hsien Rin , Wenhua Jingshen Yixue de Zengwu: Cong Taiwan dao Riben [*The Gift of Cultural Psychiatry: From Taiwan to Japan*] (Taipei: PsyGarden, 2007): 23 .

68

Howard Chiang , ‘Translating Culture and Psychiatry across the Pacific: How Koro Became Culture-bound’, History of Science, 53, 1 (2015): 102–19.

69 Wang, *op. cit.* (note 37).

70

Hsien Rin , ‘Koro: A Consideration on Chinese Concepts of Illness and Case Illustrations’, Transcultural Psychiatric Research, 15 (1963): 23–30 ; Hsien Rin , ‘A Study of Aetiology of Koro in Respect to the Chinese Concept of Illness’, International Journal of Social Psychiatry, 11, 1 (1965): 7–13 14283568 10.1177/002076406501100101.

71

Yen-Huei Chang , Hsien Rin , and Chu-Chang Chen , ‘Weihanzheng wu li baogao [Report on Five Cases of Agoraphobia],’ Bulletin of Chinese Society of Neurology and Psychiatry, 1, 2 (1975): 9–13 .

72 Rin, *op. cit.* (note 67): 60–1; Koichi Ogino , Furusato Sōshitsu no Jidai [*The Era of Homelandlessness*]. Tokyo: Hokutosha, 1979 .

73

Derek Sheridan , Dominic Men-Hsuan Yang , and Wen-liang Tseng (eds.): Studying Taiwan before Taiwan Studies: American Anthropologists in Cold War Taiwan (Taipei: Academia Sinica, 2024).

74

Tsung-Yi Lin , ‘Psychiatry in Taiwan: Past, Present and Future’, Chinese Psychiatry, 6, 1 (1992): 3–9 , on p.6.

75 Sheng-Ji Ye, *Ye Shengji Riji* [*The Diary of Sheng-Ji Ye*]. 12 December 1946 (Retrieved 21 August 2024 from https://taco.ith.sinica.edu.tw/tdk/%E8%91%89%E7%9B%9B%E5%90%89%E6%97%A5%E8%A8%98).

76 Shu Ye, *Bingli Sasan Nian* [Thirty-Three Years of Pathology] (Taipei: Zhuanji Wenxue Publishing, 1970).

77 Harry Yi-Jui Wu, ‘Transnational Trauma: Trauma and Psychiatry in the World and Taiwan, 1945-1995’ (unpublished PhD thesis: University of Oxford, 2012).

78 Erin Wu, *Taiwan Jingshen Yiliao de Kaituozhe: Yeh Eng-Kung Chuanji* [*A Pioneer of Taiwanese Psychiatry: The Biography of Eng-Kung Yeh*] (Taipei: PsychGarden, 2005): 347f; Hou-Chang Chiu et. al., *Jianchi: Taiwan Shenjing Yixue de Tuohuangzhe Hung Tsu-Pei Jiaoshou* [*Perseverance: Professor Tsu-Pei Hung, a Pioneer of Neurology in Taiwan*] (Taipei: Asian Culture Publishing, 2014); Chen, *op. cit.* (note 31).

79

Arthur Kleinman , The Soul of Care (New York: Viking, 2019): 71–2.

80 Sheridan, Yang, and Tseng, *op. cit.* (note 73).

81 Rin, *op. cit.* (note 30): 5; Chin-Ching Chen, Tsui-Lien Chen, Ching-Hsuan Su, Chun-Ying Wu, and Cheng-Hui Lin, *Zhengzhi Dangan Hui Shuohua Ziyou Shidai de Gongmin Zhinan Shi* [*Political Archives Speak: A Citizen’s Guide to the Era of Freedom*] (Taipei: Spring Hill Publishing, 2021); Yi-Jui Wu, ‘Dujuanwo li de Queniao: Zenme Kan Jingshenyixue de Zhengzhi Lanyong Shi [The Cuckoo in the Nest: Unraveling the History of Political Exploitation in Psychiatry]’, *Kám-Á-Tiàm Forum of History* (Retrieved 16 April 2024 from https://kamatiam.org/%E6%9D%9C%E9%B5%91%E7%AA%A9%E8%A3%A1%E7%9A%84%E9%9B%80%E9%B3%A5%E6%80%8E%E9%BA%BC%E7%9C%8B%E7%B2%BE%E7%A5%9E%E9%86%AB%E5%AD%B8%E7%9A%84%E6%94%BF%E6%B2%BB%E6%BF%AB%E7%94%A8%E5%8F%B2/).

82

Harry Yi-Jui Wu , ‘Psychiatrists’ Agency and their Distance from the Authoritarian State in Post World-War II Taiwan’, History of Psychology, 23, 4 (2020): 351–7032969674 10.1037/hop0000174.

83 Howard Nichlos Higginbotham, ‘Delivery of Mental Health Services in Three Developing Asian Nations: Feasibility and Cultural Sensitivity of “Modern Psychiatry”’ (unpublished PhD thesis: University of Hawaii, 1979): 118–19.

84

Eng-Kung Yeh , ‘Social Psychiatry in Taiwan (Formosa)’, in Jules H. Masserman and J. L. Moreno (eds.), Progress in Psychotherapy. Vol. IV. Social Psychotherapy (New York: Grune & Stratton, 1959): 306–9.

85

Tsung-Yi Lin , ‘Evolution of Mental Health Programme in Taiwan’, American Journal of Psychiatry, 117, 11 (1961): 961–71, on p.96813762211 10.1176/ajp.117.11.961.

86

Tsung-Yi Lin , Tsu-Pei Hung , Chiung-Ming Chen , and Tse-Ching Hsu , ‘Zhongguoren doushi zhumin zhi gaoxueyazheng yanjiu diyi baogao: Xueya pingjunzhi zhi tongjixue de kaocha [Studies on Hypertension among Urban Chinese in Taiwan (Formosa) 1. Mean Blood Pressure Readings]’, Journal of Formosan Medical Association, 55, 4 (1956): 150–9.

87

Tsung-Yi Lin , Tsu-Pei Hung , Chiung-Ming Chen , Tae-Ching Hsu , and Kung-Pei Chen , ‘A Study of Normal and Elevated Blood Pressures in a Chinese Urban Population in Taiwan (Formosa)’, Clinical Science, 18, 2 (1959): 301–1214417023 .

88

Tsu-Pei Hung , ‘Taiwan toshi kumin no kōketsuatsu hassei hindo ni kansuru kenkyū: Tokuni aya na Tsuta-sei, minzoku, shakai shūdan-kan ni okeru hindo no hikaku kenkyū [A Study on the Prevalence of Elevated Blood Pressure among Urban Chinese in Taiwan: Comparative Studies of Frequencies, Especially between Different Genders, Ethnicities, and Social Groups’, Japanese Circulation Journal, 25, 10 (1961): 1084–100.

89

Leon J. Saul , ‘Hostility in Cases of Essential Hypertension’, Psychosomatic Medicine, 1, 1 (1939): 153–61.

90 Yeh, *op. cit.* (note 84): 308.

91 Lin, *op. cit.* (note 85): 970.

92

Hunter Heyck and David Kaiser , ‘Introduction’, Isis, 101, 2 (2010): 362–620718276 10.1086/653097; Mark Solovey , ‘Cold War Social Science: Spector, Reality, or Useful Concept?’, in Mark Solovey and Hamilton Cravens (eds.), Cold War Social Science: Knowledge Production, Liberal Democracy, and Human Nature (New York: Palgrave Macmillan, 2012): 1–22 ; Solovey and Dayé, *op. cit.* (note 21).

93

Wen-Shing Tseng and Jing Hsu , ‘Chinese Culture, Personality Formation and Mental Illness’, International Journal of Social Psychiatry, 16, 1 (1970): 5–24, on p. 1310.1177/0020764070016001015408071.

94

Wen-Shing Tseng , Yige Rensheng, Sanzhong Wenhua: Zhongguo, Riben, Meiguo Wenhua dui Renge Xingcheng de Ziwo Fenxi [*One Life, Three Cultures: The Self-Analysis of the Impact of Chinese, Japanese, and American Cultures on My Personality Formation*] (Taipei: Psy Books, 2010).

